# Clinical–pharmaceutical assessment of medication CDSS alerts: content appropriateness and patient relevance in clinical practice

**DOI:** 10.3389/fphar.2025.1510425

**Published:** 2025-03-07

**Authors:** Jacqueline Bauer, Marika Busse, Sonja Koch, Marina Schmid, Julia Sommer, Martin F. Fromm, Frank Dörje

**Affiliations:** ^1^ Pharmacy Department, Universitätsklinikum Erlangen and Friedrich-Alexander-Universität Erlangen-Nürnberg, Erlangen, Germany; ^2^ Institute of Experimental and Clinical Pharmacology and Toxicology, Friedrich-Alexander-Universität Erlangen-Nürnberg, Erlangen, Germany; ^3^ FAU NeW – Research Center New Bioactive Compounds, Friedrich-Alexander-Universität Erlangen-Nürnberg, Erlangen, Germany

**Keywords:** clinical pharmacists, clinical decision support system, medication safety, overalerting, interprofessional collaboration

## Abstract

**Background:**

Clinical pharmacy services and clinical decision support systems (CDSSs) are increasingly implemented to optimize medication safety. However, risks as overalerting can limit these benefits. Therefore, the Meona medication CDSS was interprofessionally evaluated and locally configured prior to implementation at Erlangen University Hospital.

**Aim:**

We aimed to analyze the displayed CDSS alerts and to evaluate the content appropriateness and patient relevance of CDSS alerts in a hospital with established clinical ward pharmacists. Furthermore, we characterized pharmaceutical interventions triggered by CDSS and CDSS-independent interventions.

**Methods:**

Pseudonymized clinical data of 160 patients from four clinical departments were prospectively included once between days 1 and 3 after hospital admission to analyze the frequency, type, and severity of the displayed CDSS alerts. All severe and “duplicate prescription” CDSS alerts were evaluated regarding their content appropriateness and patient relevance by clinical pharmacists using the four-eyes principle. For patient-relevant CDSS alerts, clinical ward pharmacists intervened during weekly ward rounds. All pharmaceutical interventions, including CDSS-independent interventions, were documented in ADKA-DokuPIK by recording reason, acceptance rate, and severity.

**Results:**

In total, 1,799 CDSS alerts (median 9.0/patient) were displayed. Of those, 33.9% (609/1,799) were classified as severe by Meona. Clinical pharmacists validated 647 CDSS alerts (609 severe and 38 “duplicate prescriptions”). Only 82.7% (535/647) were rated as content appropriate, of which 19.6% (105/535) were classified as patient relevant. The clinical ward pharmacists recorded 244 interventions in 150 patients discussed during rounds (1.6/patient). CDSS-independent interventions by clinical ward pharmacists (158/244, 64.8%) were significantly more frequent compared to pharmaceutical interventions triggered by the CDSS (86/244, 35.2%). (p = 0.0002). The acceptance rate of interventions was 92.2% (225/244). The most common severity category was C (error occurred, no harm).

**Conclusion:**

Despite the locally customized medication CDSS, a high number of CDSS alerts were displayed. Interestingly, we still observed content-inappropriate CDSS alerts defined by pharmaceutical validation. The majority of CDSS alerts with appropriate content were rated not patient relevant in clinical practice and could be considered as overalerting. Our results highlight that a CDSS can support healthcare professionals but underline (1) the continuing need for clinical pharmacists to improve medication safety by interpreting CDSS alerts and performing comprehensive medication reviews and (2) the further need for CDSS improvements.

## 1 Introduction

Over the last 2 decades and since “To Err is Human” was published, several concepts (e.g., clinical pharmacy services, electronic prescribing, and clinical decision support systems) have been developed to optimize medication and patient safety. (Institute of Medicine US Committee on Quality of Health Care in America, 2000).

Clinical pharmacy services include various areas of activity, such as medication reconciliations and reviews, ward rounds, antibiotic stewardship programs, and patient training (Fernandes et al., 2015; Dreischulte et al., 2022). Multiple studies showed that medication reconciliations based on the best possible medication history in combination with medication reviews reduced medication errors (ME) (Mekonnen et al., 2016; Manias et al., 2020; Nguyen et al., 2020; Mengato et al., 2023). Furthermore, performing medication reviews and participating in ward rounds can contribute to the reduction of ME and, therefore, improve medication safety (Kaboli et al., 2006; Mansur, 2016; Blassmann et al., 2018; Kiesel et al., 2022). Clinical pharmacists play an important role as members of the interprofessional team for patient care in several countries worldwide. In Germany, clinical pharmacy services are expanding but have not been established nationwide (Schulz et al., 2021).

Electronic medical records (EMR) can include computerized physician order entry (CPOE) and clinical decision support systems (CDSSs). Both CPOE and CDSSs have the potential to reduce ME and subsequently optimize medication safety (Bates et al., 1999; Garg et al., 2005; Vélez-Díaz-Pallarés et al., 2018). However, the implementation of CPOE and CDSSs is associated with risks such as overalerting and nonacceptance among the end users (Ammenwerth et al., 2008; Wolfstadt et al., 2008; van der Sijs et al., 2009; Nuckols et al., 2014; Ranji et al., 2014; Jia et al., 2016; Nanji et al., 2018; Abell et al., 2023). Thus, the implementation needs to be well planned and the performance monitored in clinical practice (Ammenwerth et al., 2014; The Office of the National Coordinator for Health Information Technology, 2016; Wasylewicz and Scheepers-Hoeks, 2019). As data on the pre-implementation evaluation of CDSSs were scarce, we previously developed an algorithm for the interprofessional validation of a medication CDSS and performed the evaluation for Meona, a commercial CPOE and CDSS (Bauer et al., 2024). We focused on evaluating the general functionalities of the CDSS as well as on performing a technical and content-related validation of single elements of the CDSS (e.g., drug–drug interactions). As a result of this prior evaluation, we decided interprofessionally which elements of CDSS should be used and how they should be configured. Thus, we implemented a thoroughly customized medication CDSS into clinical practice at Erlangen University Hospital (Bauer et al., 2024).

Previous studies on medication safety investigated the effect of clinical ward pharmacists in combination with CPOE/CDSS. These studies showed that clinical ward pharmacists can identify drug-related problems in addition to the implemented CPOE/CDSS (Bedouch et al., 2009; Bedouch et al., 2012; Zaal et al., 2013; Cornu et al., 2014; Berger et al., 2022; Seiberth et al., 2022). However, only a few studies performed a pharmaceutical validation of the displayed CDSS alerts in real-world clinical settings (Zaal et al., 2013; Cornu et al., 2014).

The present investigation is the first to describe in detail the synergy between a medication CDSS and clinical ward pharmacist activities. For this reason, and because Meona is one of the most widely used CPOE/CDSS in Germany but lacks evaluation data, we conducted this study within a large German university hospital. We aimed to examine the performance of our customized CDSS after implementation in clinical practice by analyzing the frequency, type, and severity of the displayed CDSS alerts and to evaluate the content appropriateness and patient relevance of the displayed CDSS alerts. A further objective of this investigation was to characterize both the interventions performed by the clinical ward pharmacists supported by the CDSS and CDSS-independent interventions according to their reason, level of severity, and acceptance rate.

## 2 Material and methods

### 2.1 Setting

The Erlangen University Hospital comprises 50 clinical departments and interdisciplinary centers and provides 1,462 beds (Universitätsklinikum Erlangen Number and facts, 2025). In this investigation, pseudonymized clinical data from four clinical departments were included: Medicine 1, Medicine 4, Trauma Surgery-Orthopedics, and Vascular Surgery. Medicine 1 includes several medical disciplines, such as gastroenterology, pneumology, and endocrinology, and three normal wards. Medicine 4 consists of one normal ward, and the medical disciplines are nephrology and hypertension. The Trauma Surgery-Orthopedics and the Vascular Surgery departments treat their patients on two and one normal wards, respectively. In June 2020, Meona (Mesalvo Freiburg GmbH, 2023) was implemented as an EMR step-by-step in all normal wards at Erlangen University Hospital.

### 2.2 Software

Meona (Mesalvo Freiburg GmbH, 2023) is a commercially available EMR and includes a CPOE with an integrated medication CDSS. The medication CDSS operates based on rules rather than using machine learning or neuronal networks and presents interruptive as well as passive/on-demand alerts. The CDSS comprises 19 Medication-Safety-Validators (see Supplementary Figure S1), each addressing a different topic (e.g., drug–drug interactions and duplicate prescriptions). Before implementing the CDSS into clinical practice, an interprofessional team evaluated the CDSS and its included Medication-Safety-Validators concerning the general functionalities, technical, and content-related limitations (Bauer et al., 2024). Through this preceding work, new developments and improvements of the CDSS were achieved, such as a new configuration option (“only-PULL-modus”) (Bauer et al., 2024). If this configuration is set, all CDSS alerts are only displayed on demand and must be retrieved proactively by clicking the check button. The other possible configuration options are the “PUSH-(&PULL)-modus” (interruptive CDSS alerts during medication prescription and presenting the same CDSS alerts in the check button) and “OFF-modus.” In general, CDSS alerts displayed in Meona are stratified into three severity grades: severe, medium, and low. As a result of the evaluation process, we decided to use four Medication-Safety-Validators in the “PUSH-(&PULL)-modus,” three Medication-Safety-Validators in “only-PULL-modus,” and 12 Medication-Safety-Validators in the “OFF-modus.” For details, a visualization and the configuration of the Medication-Safety-Validators at Erlangen University Hospital are shown in Supplementary Figures S1, S2. The check button is integrated into the patient chart (see Supplementary Figure S2B).

### 2.3 Clinical pharmacy services at Erlangen University Hospital

Clinical pharmacy services have been established at Erlangen University Hospital since 2010. All clinical ward pharmacists are specialized for their designated medical department and accompany ward rounds once a week at these clinical departments. The clinical ward pharmacists perform different additional tasks depending on each clinical department. For instance, a medication reconciliation service at patient admission in combination with advanced medication review is conducted in all surgical departments. Furthermore, clinical ward pharmacists participate in an interprofessional, weekly antibiotic stewardship visit in Medicine 1 and 4 and provide intensified training for transplant patients treated with immunosuppressants in Medicine 4. In all wards, the validated German database ADKA-DokuPIK is used to document pharmaceutical interventions (ADKA, 2025; Ihbe-Heffinger et al., 2019).

### 2.4 Structural analysis of the displayed CDSS alerts

**FIGURE 1:**
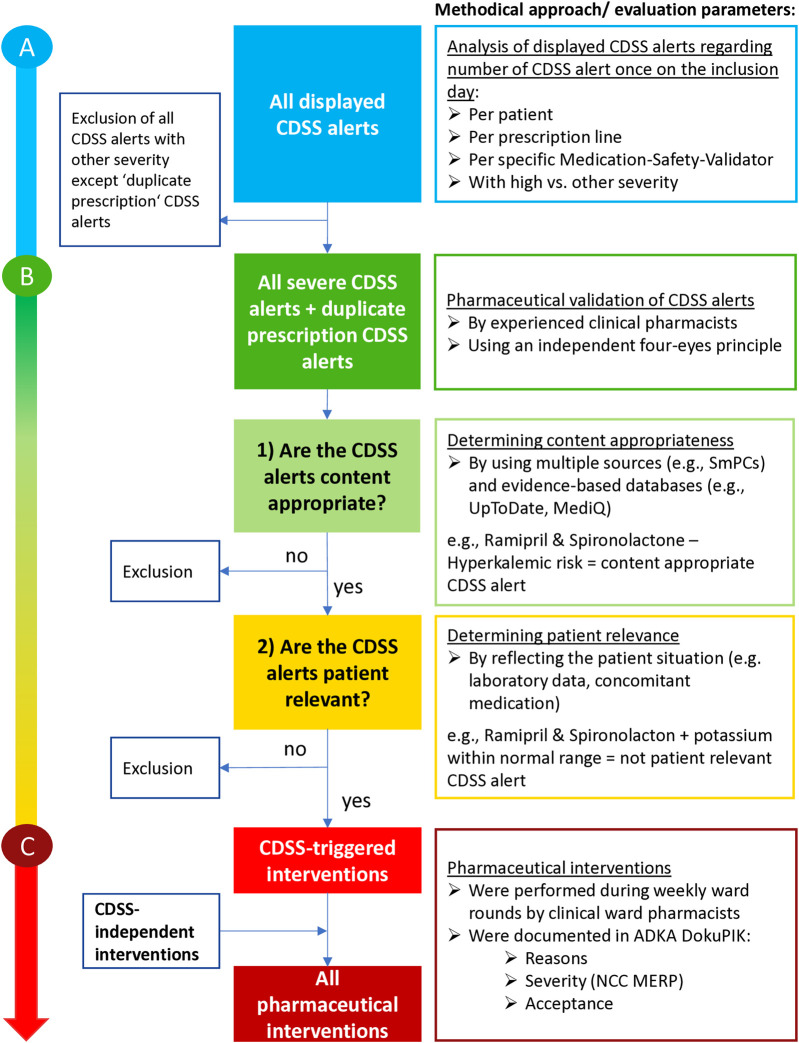
Methodological approach for the evaluation of all displayed CDSS alerts shown by using the check button **(A)** for pharmaceutical validation of the CDSS alerts **(B)** and for pharmaceutical interventions, if necessary **(C)**. Abbreviations: CDSS, clinical decision support system; SmPC, Summary of product characteristics; ADKA-DokuPIK, database for reporting medication errors and interventions in Germany; NCC MERP, National Coordinating Council for Medication Error Reporting and Prevention.

In this analysis, we performed a point-prevalence study by prospectively including the pseudonymized clinical data of 40 patients from each of the four clinical departments between July 2022 and July 2023. We consecutively included the clinical data of all patients who met the inclusion criteria (at least two drugs prescribed and hospital admission between 1 and 3 days ago) over several weeks until 40 patients were included in one department. Thereafter, the inclusion phase in the next department was started. As inclusion days, we always chose the ward round day of the designated clinical ward pharmacists. We assessed the CDSS alerts displayed in the check button at one single time point regarding the number, type, and severity of CDSS alerts. In addition, we reported the number of prescription lines for each patient (see Figure 1A).

### 2.5 Pharmaceutical validation of CDSS alerts

Due to the high number of expected CDSS alerts, we performed the pharmaceutical validation only for CDSS alerts with high severity defined by Meona and for all displayed “duplicate prescriptions” CDSS alerts. This approach was chosen as “duplicate prescriptions” CDSS alerts are never classified with high severity in Meona. The pharmaceutical validation was independently performed using a four-eyes principle by the study clinical pharmacist and the clinical ward pharmacists responsible for each clinical department. The median professional experience of clinical pharmacists was 6.0 years (range 4.0–18.0). The study clinical pharmacist had experience in attending ward rounds without specialized experience in the four medical departments. The evaluation was carried out on two consecutive levels: 1) Is the content of the displayed CDSS alert appropriate? and 2) Is the CDSS alert patient relevant? (Figure 1B). For example, the CDSS alert of the drug–drug interaction between candesartan and spironolactone is content appropriate regarding its hyperkalemic risk. However, the CDSS alert was rated not patient relevant if the patient had potassium levels within the normal range. Multiple valid sources [e.g., German summaries of product characteristics (SmPCs) and evidence-based medical databases (e.g., UpToDate, Martindale, and crediblemeds) or drug–drug interaction checks (LexiInteract; MediQ)] were used to determine content appropriateness of the displayed CDSS alerts. If the content appropriateness was assessed differently in the multiple, evidence-based sources, a clinical–pharmaceutical discussion and weighting between the responsible clinical ward pharmacist and study clinical pharmacist was conducted. Patient relevance was assessed by considering the current individual situation (e.g., laboratory data and concomitant medication). For more details and examples for the validation of appropriateness and patient relevance, see Supplementary Table S1. If a CDSS alert was classified differently within the four-eyes principle, the two responsible clinical pharmacists discussed the situation and, if necessary, consulted a third independent clinical pharmacist. If a CDSS alert was either classified as content inappropriate or not patient relevant, the reasons for this decision were recorded.

### 2.6 Pharmaceutical interventions to improve medication safety

The responsible clinical ward pharmacist discussed all patient-relevant CDSS alerts with physicians during regular weekly ward rounds (Figure 1C). If an intervention was classified as patient relevant, but no intervention was performed, the underlying reason was documented. Pharmaceutical interventions triggered by CDSS and all CDSS-independent interventions carried out within the comprehensive medication review by the clinical ward pharmacists were documented in ADKA-DokuPIK 1.0 (ADKA, 2025). The CDSS-triggered interventions were marked in the database in order to allow a comparative evaluation between the two intervention types. The documentation of the pharmaceutical interventions in ADKA-DokuPIK enabled an analysis of the frequency and reasons for interventions, involved drugs, acceptance rate, and severity according to NCC MERP (1998).

### 2.7 Data analysis

The present investigation was conducted in accordance with the Bavarian Hospital Act (Bayerisches Krankenhausgesetz, BayKrG) Article 27 (4). Because only data from the routine care of clinical pharmacy services was used, no separate approval from the ethics committee was required. The data collected in this study were documented in a pseudonymized form. Data analyses were performed with Microsoft Excel^®^. All results are presented anonymously. Descriptive statistical analyses were performed in GraphPad Prism^®^ with a 95% confidence interval. Continuous variables were compared using the Wilcoxon rank sum test (e.g., number of interventions). Fisher’s exact test was used to compare categorical variables (e.g., severity).

## 3 Results

### 3.1 Structural analysis of the displayed CDSS alerts

Overall, clinical data of 160 patients were included. Table 1 presents the patient characteristics of all patients. Median age was 69.0 years (range 11.0–105.0), and 92.5% (148/160) of the patients were taking long-term medications. In the median, 9.0 medication prescriptions per patient were recorded (range 2.0–32.0). Most prescription lines were documented in the median for patients of Medicine 4 (15.0) and Vascular Surgery (14.5). Approximately half of the patients (46.9%, 75/160) had a renal impairment (eGFR<60 mL/min) or were on dialysis. Patient characteristics for each clinical department are displayed in Supplementary Table S2.

**TABLE 1:** Patient characteristics.

Patient characteristics	Number of patients (N = 160)
Age in years median (range)	69 (11.0–105.0)
Age >65 years number (%)	95 (59.4)
Prescription lines per patient median (range)	12.0 (2.0–32.0)
Use of long-term medication number (%)	148 (92.5)
Polymedication^a^ number (%)	104 (65.0)
Patients with renal impairment^b^ number (%)	
eGFR ≥60 mL/min/1.73 m^2^	85 (53.1)
eGFR 59–30 mL/min/1.73 m^2^	31 (19.4)
eGFR <30 mL/min/1.73 m^2^	28 (17.5)
Dialysis	16 (10.0)

^a^ Polymedication is defined as the use of at least five long-term medications.

^b^ Using CKD-EPI as an estimation for the GFR (glomerular filtration rate).

**FIGURE 2:**
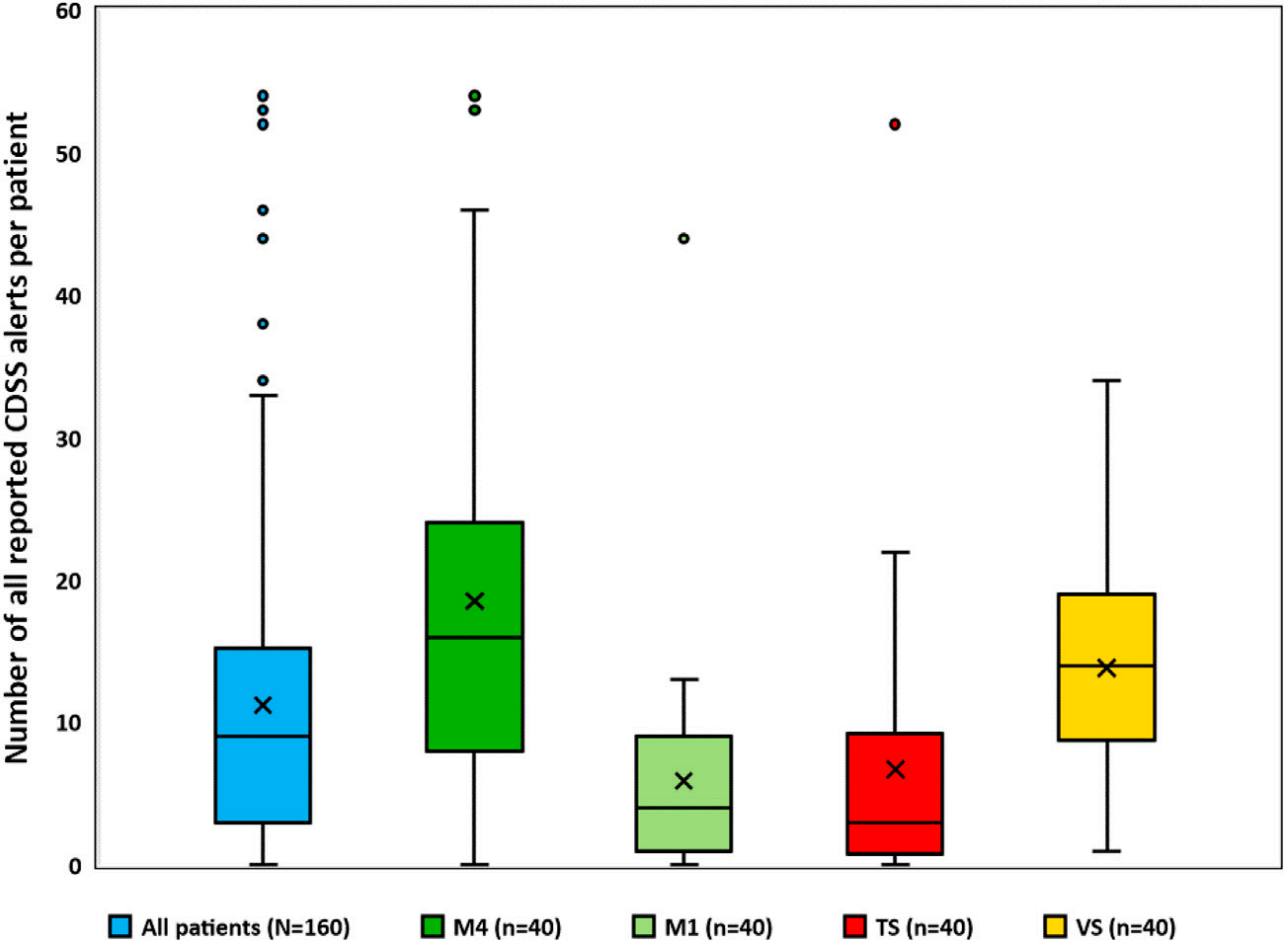
Number of all reported CDSS alerts for all patients and for each clinical department. Box plots with x representing the mean, the horizontal line representing the median, and whiskers based on the 1.5 IQR (interquartile range) value. Abbreviations: M4, Medicine 4; M1, Medicine 1; TS, trauma surgery; VS, vascular surgery; CDSS, clinical decision support system.

In total, 1,799 CDSS alerts were presented for the 160 patients (in median 9.0 CDSS alerts/patient). Of those, 33.9% (609/1,799) were classified as severe by Meona. [see Table 2 (total patient collective); Supplementary Table S3 (each clinical department)] Figure 2 indicates the number of CDSS alerts per patient for each clinical department and the total patient collective. The highest median number of CDSS alerts was found in Medicine 4 (16.0 CDSS alerts/patient) and the lowest in Trauma Surgery (3.0 CDSS alerts/patient).

**TABLE 2:** Total number of CDSS alerts per patient and per prescription lines.

	Total alerts (N = 1799)
Number of CDSS alerts/patient median (range)	9.0 (0.0–54.0)
Number of CDSS alerts/prescription line	0.9
Number of CDSS alerts with high severity	609
CDSS alerts with high severity/patient median (range)	3.0 (0.0–19.0)

For details (differences between the different clinical departments), see Supplementary Table S3. Abbreviation: CDSS, clinical decision support system.

**FIGURE 3:**
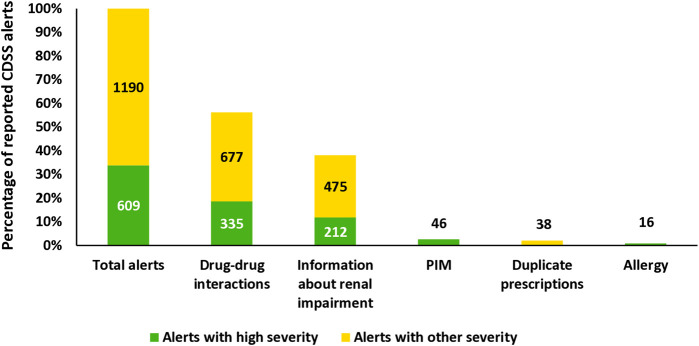
Percentage of all displayed CDSS alerts (high severity or other severity) in total and for each specific Medication-Safety-Validator. The Medication Safety-Validator “Frequency of administration” was excluded because no CDSS alerts were displayed in this investigation. Abbreviation: PIM, potential inadequate medication in the elderly.

With a proportion of 56.3% of the total CDSS alerts (1.012/1.799), the majority of the CDSS alerts were caused by the Medication Safety Validator “drug-drug interactions” (see Figure 3). The second most frequent CDSS alerts were attributed to the Medication-Safety-Validator “information about renal impairment” (687/1,799, 38.2%), while CDSS alerts from all other Medication-Safety-Validators accounted only for a minority. The Medication-Safety-Validator “frequency of administration” did not cause any CDSS alert in this investigation. In total, 189 different drug–drug interactions were registered (e.g., 22× “buprenorphine & ondansetron,” 13× “buprenorphine & tramadol,” and 12× “ondansetron & tramadol”). The Medication-Safety-Validator “information about renal impairment” displayed CDSS alerts for 108 different drugs (e.g., 33× enoxaparin, 22× acetylsalicylic acid low dose, and 15× candesartan).

### 3.2 Pharmaceutical validation of CDSS alerts

**FIGURE 4:**
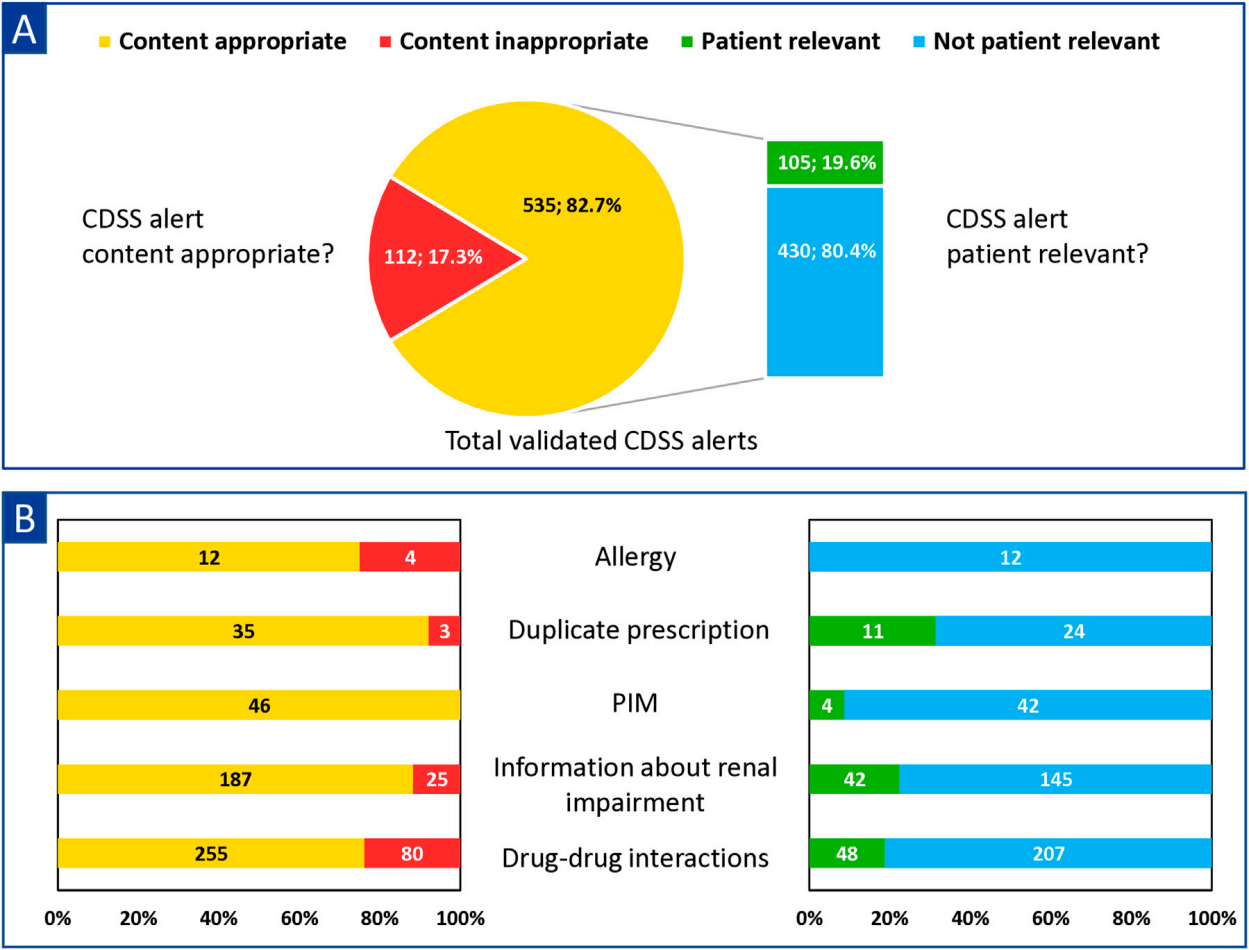
Numbers and proportions of content appropriate and patient relevant and CDSS alerts for **(A)** the total validated CDSS alerts [N = 647 (severe and all “duplicate prescription” CDSS alerts)] and **(B)** each Medication-Safety-Validator. Abbreviations: PIM, potential inadequate medication in the elderly; CDSS, clinical decision support system.

Two experienced clinical pharmacists validated a total of 647 CDSS alerts with the four-eyes principle, including 609 CDSS alerts with high severity and 38 “duplicate prescriptions” CDSS alerts. The assessment of clinical appropriateness showed that 535/647 (82.7%) CDSS alerts were rated as content appropriate (Figure 4A). 17.3% (112/647) of the validated CDSS alerts were classified as content inappropriate (e.g., incorrect or not evidence-based CDSS alerts with regard to the current state of scientific knowledge). Most common CDSS alerts classified as content inappropriate were attributable to “drug–drug interactions,” “allergy,” and “information about renal impairment” (Figure 4B). As examples, the following drug–drug interactions were classified as content inappropriate: *28× drugs with very low amounts of potassium* (e.g., macrogol and potassium chloride - Movicol^®^, electrolyte solution - Jonosteril^®^) *& drugs with hyperkalemic risk* (e.g., ramipril, candesartan, and spironolactone), *25*× *combination of two drugs with only possible risk/conditional risk for QT-prolongation as a severe CDSS alert* (e.g., tramadol and buprenorphine), and *8*× *bleeding risk in the combination of “dipyrone & clopidogrel.”* Of the 25 content-inappropriate “information about renal impairment” CDSS alerts, 22 concerned acetylsalicylic acid low dose, which is not recommended in eGFR <50 mL/min/1.73 m^2^, according to Meona. Differences in the percentages of content appropriateness were noted between the clinical departments (80.2%–86.3%) (see Supplementary Figure S3A).

Of the 535 CDSS alerts rated content appropriate, 19.6% (105/535) were classified as patient relevant with the four-eyes principle in the second validation level (see Figure 4A). Within the different alert types, CDSS alerts regarding “duplicate prescriptions” and “information about renal impairment” were most frequently classified as patient relevant, whereas “allergy” CDSS alerts were never found to be patient relevant. (see Figure 4B). The proportion of patient-relevant CDSS alerts among the clinical departments differed highly (9.1%–40.9%) (for details, see Supplementary Table S3B).

### 3.3 Pharmaceutical interventions to improve medication safety

Of 1,799 displayed CDSS alerts, 105 (5.8%) were rated content appropriate and patient relevant and, therefore, met the criteria to be addressed by the clinical ward pharmacists during ward rounds. No intervention was performed for eight of 105 (7.6%) CDSS alerts because the involved medication had already been discontinued in the patient’s discharge medication or intervention was not feasible during ward rounds (e.g., intensive patient education). In a few cases, several CDSS alerts regarding one patient led to the same intervention by the clinical ward pharmacists [e.g., several drug–drug interactions with QT-prolonging drugs led to the recommendation of an electrocardiogram (ECG) control], and, therefore, only one intervention was documented in ADKA-DokuPIK. These cases are shown as examples in Supplementary Table S4. This approach resulted in 86 CDSS-triggered interventions by clinical ward pharmacists.

**FIGURE 5:**
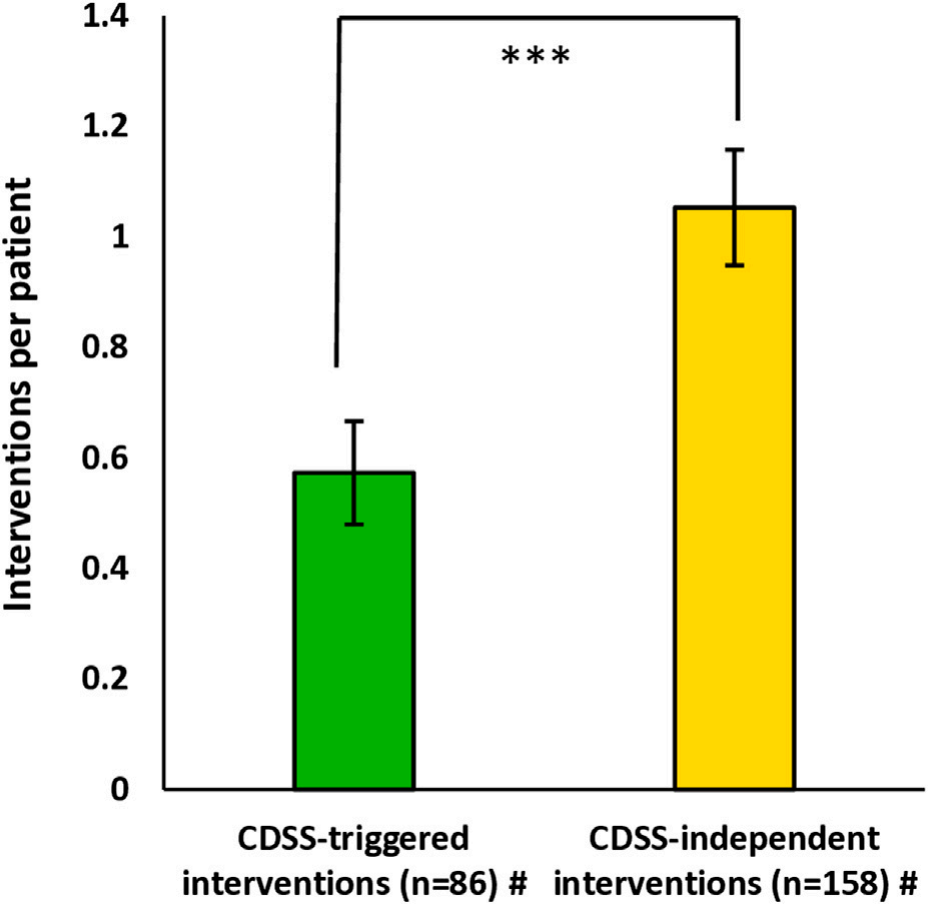
CDSS-triggered and CDSS-independent interventions performed by the clinical ward pharmacists per patient. Data are shown as mean+/-standard error of the mean. Significance was tested with the Wilcoxon rank sum test and a significance level of α = 0.05. ^#^ For 150 patients discussed during ward rounds, 10 patients could not be visited due to discharge or surgery. Abbreviation: CDSS, clinical decision support system.

In total, 244 interventions were carried out by the clinical ward pharmacists for 150 patients discussed during ward rounds (1.6/patient). Ten patients could not be discussed interprofessionally due to premature discharge or surgery. A significantly higher number of 158 interventions (1.1/patient) were CDSS-independent interventions identified by the clinical ward pharmacists during comprehensive medication review compared to 86 of 244 interventions (0.6/patient) triggered by CDSS (p = 0.0002) (Figure 5). The mean number of interventions differed highly between the clinical departments (0.8–2.4 interventions/patient); see Supplementary Table S5. The most common reasons, according to ADKA-DokuPIK, for CDSS-triggered interventions were “dose” [e.g., failure to adjust dose for organ dysfunctions (40/86, 46.5%)] and “interaction” (21/86, 24.4%). For the CDSS-independent interventions, the most common reasons were “drugs” [e.g., (clear) indication but no drug prescribed (80/158, 50.6%)] and “dose” (67/158, 42.4%); for details, see Supplementary Figure S4. All possible reasons according to ADKA-DokuPIK categories with their absolute frequencies for the interventions are shown in Supplementary Table S6.

**FIGURE 6:**
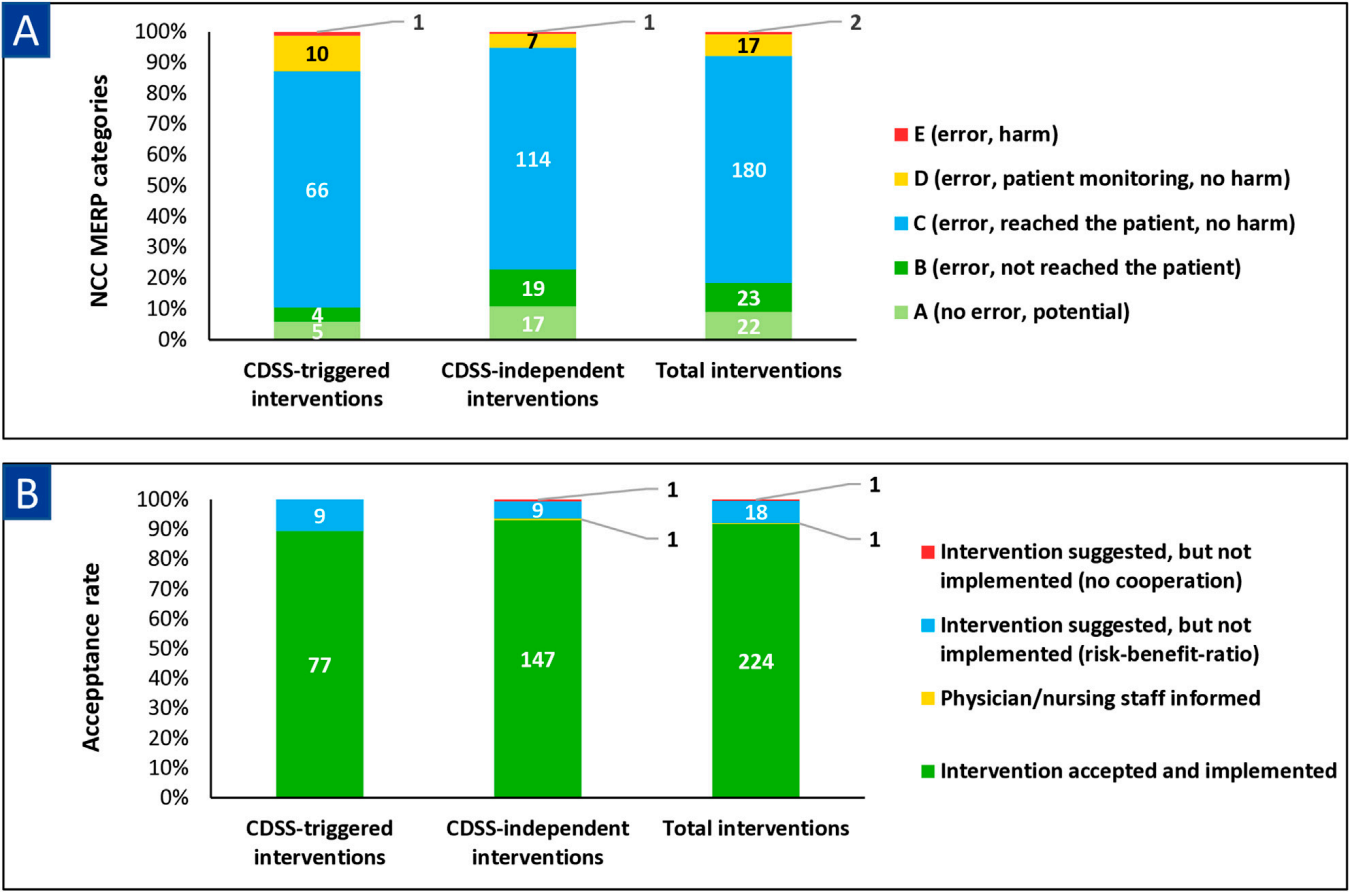
Classification of **(A)** pharmaceutical interventions according to NCC MERP and **(B)** acceptance rates of pharmaceutical interventions stratified for CDSS-triggered interventions, CDSS-independent interventions, and total interventions. Abbreviations: CDSS, clinical decision support system; NCC MERP, National Coordinating Council for Medication Error Reporting and Prevention. Note: NCC MERP categories: A: no actual, but potential error; B: error occurred but did not reach the patient; C: error occurred and reached the patient, but without harm; D: error reached the patient and required monitoring/intervention to preclude harm; E: error contributed to temporary patient harm and required intervention.

NCC MERP category C (error occurred and reached the patient, but without harm) was the most common severity level for the CDSS-triggered interventions (66/86, 76.7%) and as well for the CDSS-independent interventions (114/158, 72.2%) (Figure 6A). For the CDSS-independent interventions, categories A (no actual error occurred, but potential) and B (error occurred, but did not reach the patient) were significantly more frequent compared to categories C–E than for the CDSS-triggered interventions (p = 0.0238). The acceptance rate of the performed interventions was 92.2% (225/244) and did not differ significantly (p = 0.3174) between interventions triggered by the CDSS and CDSS-independent interventions (Figure 6B).

## 4 Discussion

To the best of our knowledge, we performed the first investigation to determine the performance of the Meona-CDSS in clinical practice. We did not set many inclusion criteria to obtain clinical data of a broad and real-world representing patient collective. Furthermore, no prospective studies in Germany assessed the performance of a commercial, integrated CDSS in clinical practice by using a pharmaceutical validation of the CDSS alerts (see Figure 1). We evaluated our customized medication CDSS (see Supplementary Figure S1) within four different clinical departments at Erlangen University Hospital by analyzing the number, type, and severity level of shown CDSS alerts. Subsequently, the content appropriateness and patient relevance of the displayed CDSS alerts were determined by clinical pharmacists using a four-eyes principle. The clinical ward pharmacists performed interventions for all patient-relevant CDSS alerts, and the CDSS-independent identified drug-related problems during regular ward rounds and documented these in ADKA-DokuPIK. Thus, reasons, severity using NCC MERP, and acceptance rate of pharmaceutical interventions were evaluated (see Figure 1).

In general, we registered 1,799 CDSS alerts in total (in median 9.0/patient) and 609 severe CDSS alerts (in median 3.0/patient) (see Table 2). The best comparable study by Zaal et al. found 2.7 CDSS alerts/patient using a CDSS system based on the Dutch national drug database with interruptive alerts (overdose, allergies, duplicate therapy) and on-demand alerts (drug–drug interactions) (Zaal et al., 2013). Mc Coy et al. observed 1.6 CDSS alerts on acute kidney injury per patient displayed by a locally developed CDSS (McCoy et al., 2012). Russmann et al. reported 15.5 CDSS alerts per patient using two different CDSSs [PharmaVista (drug–drug interactions, therapeutic duplications, maximum dose, potential inadequate medication in the elderly) and MediQ (focus on drug–drug interactions)] (Russmann et al., 2023). However, a direct comparison is difficult as the study time, the methods used, and the CDSS systems are different.

The highest numbers of CDSS alerts were recorded for patients from Medicine 4 and Vascular Surgery (see Figure 2). This can be explained by the fact that most prescription lines were recorded for these clinical departments (see Supplementary Table S2), predominantly treating patients with a variety of comorbidities (e.g., diabetes, renal impairment) who required polymedication. Previous studies showed a positive correlation between the number of drugs and drug-related problems (Saedder et al., 2016; Høj et al., 2021; Jung-Poppe et al., 2022; Berger et al., 2023).

The most frequently observed types of CDSS alerts were “drug-drug interaction” and “information about renal impairment” (see Figure 3). Other studies reported similar results for the most reported type of CDSS alerts (van der Sijs et al., 2009; Zaal et al., 2013). In our investigations, it is possible that only a few “duplicate prescription” and “allergy” CDSS alerts were still displayed using the check button because these two Medication-Safety-Validators are used with the PUSH-(&PULL)-modus (see Supplementary Figure S1B). Thereby, these CDSS alerts are displayed as pop-up alerts during the medication prescription process. Physicians probably registered these CDSS alerts when prescribing a medication and adjusted their medication prescriptions accordingly.

We performed the pharmaceutical validation for all severe (609) and “duplicate prescription” (38) CDSS alerts with a two step-approach (see Figure 1). First, we determined a content appropriateness of 82.7% (535/647) among the CDSS alerts meeting our inclusion criteria (see Figure 4A). Second, we found overalerting, as only 19.6% (105/535) of the content-appropriate CDSS alerts were classified as patient relevant (see Figure 4A).

Interestingly, many of the content-inappropriate CDSS alerts in this study are based on a few recurring scenarios, such as severe “drug–drug interaction” CDSS alerts for drugs with only possible risk for QT-prolongation according to AZCERT criteria or “information about renal impairment” CDSS alerts for acetylsalicylic acid low dose. These scenarios were discussed with the manufacturer to adapt the rule-based CDSS accordingly (e.g., regarding their severity). In general, better performance of the medication CDSS can be achieved by initiating the improvements through clinical pharmacists (Cuvelier et al., 2021; Skalafouris et al., 2023).

The fact that a content-appropriate CDSS alert was often rated not patient relevant (430/535, 80.4%) was usually due to the insufficient specificity of the CDSS alerts (Fritz et al., 2012; de Wit et al., 2015; Cuvelier et al., 2021; Russmann et al., 2023; Bauer et al., 2024). In the case of “information about renal impairment” CDSS alerts, for example, the prescribed dose is not checked. A CDSS alert is triggered if the renal function is below a certain threshold regardless of the actual dose prescribed (e.g., eGFR 35 mL/min and Sitagliptin 50 mg 1-0-0-0 caused an CDSS alert, although the prescribed dose was already correct). As another example, drug–drug interactions are also displayed, even though a required action, such as dose reduction (e.g., amlodipine and max. 20 mg simvastatin) or specific administration interval (e.g., calcium and levothyroxine) had already been considered to preclude the interaction. Only a few PIM (potential inadequate medication in the elderly) CDSS alerts were rated patient relevant, due to the lack of better alternatives for a given indication in clinical practice. Our results showed that the medication CDSS should be improved to enable more efficient clinical decision support and to reduce overalerting. Improvements of the CDSS should include considering *inter alia* the actual doses prescribed, the time of administration (e.g., morning or evening), the diagnoses, as well as more laboratory data and clinical parameters such as blood pressure or pain scores.

Other studies reported that overridden rates are higher among CDSS alerts regarding drug–drug interactions, geriatric recommendations, and renal dose adjustments as opposed to alerts affecting duplicate prescription, allergy, and overdose (van der Sijs et al., 2009; Poly et al., 2020). These findings are in line with the results of our pharmaceutical validation as “duplicate prescriptions” alerts were most frequently rated as patient relevant, followed by “information about renal impairment” alerts (see Figure 4B).

Overalerting is associated with nonacceptance as well as reduced satisfaction and reliability of the CDSS in clinical practice and is frequently reported by other investigations (Zaal et al., 2013; Nanji et al., 2018; Abell et al., 2023). Most of the alerts displayed by our customized CDSS are rated as content appropriate, but the CDSS still reported many not-patient-relevant CDSS alerts with respect to the patient situation in clinical practice, leading to overalerting while using the check button (see Figure 4A). This may disturb the physicians’ efficient use of the check button. Nevertheless, with our special configuration (“only-PULL-modus”) for several Medication-Safety-Validators (see Supplementary Figure 1B), CDSS alerts were at least not displayed as popup alerts during medication prescription, which might be an effective strategy to reduce overalerting (Knight et al., 2019; Bauer et al., 2024).

In general, experience with the CDSS or other databases is required to assess the CDSS alerts in an appropriate way. This might be challenging for healthcare professionals in clinical practice. Thereby, training on handling the CDSS for all healthcare professionals, especially physicians and clinical pharmacists, should be provided (The Office of the National Coordinator for Health Information Technology, 2016; Van de Velde et al., 2018; Abell et al., 2023). However, an integrated CDSS can support healthcare professionals, especially clinical pharmacists, to improve medication safety, as more review time can be provided due to integrated clinical decision support (Urbina et al., 2021).

Our investigation showed that the clinical ward pharmacists recorded a significantly higher number of CDSS-independent interventions (1.1/patient) during their regular rounds than pharmaceutical interventions triggered by the CDSS (0.6/patient) (see Figure 5). One reason for the high number of CDSS-independent interventions by the clinical ward pharmacists could be the high level of clinical expertise, as each clinical ward pharmacist had been assigned to a specific clinical department as part of the interprofessional team for a long time. Several published studies emphasized that clinical pharmacists can additionally identify pharmaceutical interventions beyond the CDSS by performing medication reviews in different settings (e.g., a renal pharmacist consultant service or closed-loop medication management) (Bedouch et al., 2009; Bedouch et al., 2012; Zaal et al., 2013; Berger et al., 2022; Seiberth et al., 2022). One reason might be that clinical pharmacists can consider patient individual aspects (e.g., laboratory parameters, diagnoses) in their medication review, which is limited for most ruled-based medication CDSS (e.g., identifying missing drug treatment despite existing indications). Therefore, in our analysis, the causes for pharmaceutical interventions (CDSS-triggered and CDSS-independent interventions) differed between the two intervention types (see Supplementary Figure S4; Supplementary Table S6). Clinical ward pharmacists intervened much more frequently on the indication or appropriateness of a drug. These are also the most frequently cited reasons for pharmaceutical interventions in other studies (Bedouch et al., 2012; Zaal et al., 2013; Berger et al., 2022; Langebrake et al., 2022).

The severity level (see Figure 6A) of our pharmaceutical interventions according to NCC MERP is, in general, comparable to other studies (Langebrake et al., 2015; Berger et al., 2022; Langebrake et al., 2022). In detail, some studies reported severity levels A and B (no error or error did not reach the patient) more frequently, which may be due to the daily activity of the clinical pharmacists in these studies compared to our once-weekly rounds (Berger et al., 2022). In our investigation, the NCC MERP severity levels A and B occurred significantly more often for the CDSS-independent interventions than for the CDSS-triggered interventions, thus indicating that clinical ward pharmacists perform many preventive interventions (e.g., constipation prophylaxis for opioid-treated patients). On the other hand, the CDSS alerts are only displayed by using the check button when the error has usually already reached the patient (i.e., after medication prescription).

Furthermore, the acceptance rate of our performed interventions was very high (92.2%, 225/244) in total and did not differ significantly between the two intervention types (see Figure 6B). Other studies reported acceptance rates of pharmaceutical interventions between 55.0% and 91.9% (Bedouch et al., 2012; Zaal et al., 2013; Zaal et al., 2020; Berger et al., 2022; Langebrake et al., 2022). A reason for this might be that all interventions were directly communicated by clinical ward pharmacists, which seemed to be more effective than the digital communication within the EMR used in prior studies (Bedouch et al., 2012; Zaal et al., 2020).

In contrast to Bittmann et al., who analyzed the performance of their CDSS (AiDKlinik (Dosing GmbH, 2023)) by analyzing the overridden rates (Bittmann et al., 2021), we used a prospective pharmaceutical validation with a four-eyes principle. In the published literature, we could only identify one similar multi-level approach for the pharmaceutical validation of CDSS alerts (Cuvelier et al., 2021). Other studies evaluated the appropriateness of overridden popup-CDSS alerts retrospectively by clinical pharmacists or interprofessional teams (McCoy et al., 2012; Nanji et al., 2018; Van De Sijpe et al., 2022). In our investigation, the interaction between an integrated medication CDSS and a clinical ward pharmacist service was described and examined in detail for the first time in order to record and evaluate the synergies between both approaches to improve medication safety. Our investigation confirmed that customizing the CDSS by using the “only-PULL-modus” is a useful configuration option to prevent interruptive overalerting during medication prescription, as most of the displayed CDSS alerts were rated not patient relevant (Bauer et al., 2024).

As a constraint of our investigation, the findings were only collected in four clinical departments at one site and with one site-specific, customized medication CDSS. Therefore, the results cannot be transferred directly to other sites and settings. Other studies showed that clinical pharmacists could identify additional and more drug-related problems than a medication CDSS, but the number and type of CDSS alerts might be different within other CDSS and sites (Bedouch et al., 2009; Bedouch et al., 2012; Zaal et al., 2013; Seiberth et al., 2022). Data interpretation might further be limited by systemic biases resulting from local resources and might limit scalability (e.g., time burden of pharmaceutical medication reviews and clinical expertise of clinical pharmacists). Our approach to determine the performance of our customized medication CDSS has some further limitations: Due to the point-prevalence design, we assessed the CDSS alerts and pharmaceutical interventions only at one single time point and not for the whole stay of the patients. The pharmaceutical validation of the CDSS alerts was examined with the four-eyes principle, but we did not perform a Delphi approach. Furthermore, the inter-rater reliability during the independent pharmaceutical validation was not determined in a structured way. We only validated severe CDSS alerts and all duplicate prescription CDSS alerts, potentially excluding important CDSS alerts with lower severity. The validation of the CDSS alerts was solely carried out by clinical pharmacists without involving an interprofessional team during the validation. Due to the high acceptance rate of the CDSS-triggered interventions by clinical pharmacists, we can rule out overalerting of the physicians with non-patient-relevant interventions. Other studies directly involving physicians in validating the CDSS alerts also found that most of the CDSS alerts were correct in terms of form, technology, and content but were frequently not classified as patient relevant (Pfistermeister et al., 2016; Nanji et al., 2018). We did not investigate the physicians’ responses to the displayed medication CDSS alerts in this analysis, as, for example, McCoy et al. (2012) reported. Thus, it was not possible to determine whether the check button was invoked or which CDSS alerts were read or accepted.

## 5 Conclusion

This investigation showed that a locally customized medication CDSS indicated a high number of CDSS alerts per patient, a third of which were classified as severe. Although content-inappropriate CDSS alerts were still identified, the majority of the clinical pharmacist-validated CDSS alerts were rated as content appropriate. Only a minority of the content-appropriate CDSS alerts were classified as patient relevant and consequently required an intervention by clinical ward pharmacists. These results can be considered as overalerting in clinical practice and showed the need for improvements in the medication CDSS (e.g., including diagnoses and laboratory data) to enable more efficient clinical decision support. In addition, the clinical ward pharmacists carried out significantly more CDSS-independent than CDSS-triggered interventions. To conclude, a customized medication CDSS can support healthcare professionals in optimizing medication safety. However, our results underline the continuing need for clinical pharmacists to interpret the CDSS alerts in the individual patient context and to perform a comprehensive medication review to identify all drug-related problems.

## Data Availability

The original contributions presented in the study are included in the article/Supplementary Material; further inquiries can be directed to the corresponding author.
