# Supplementary material for: Clinical–pharmaceutical assessment of medication CDSS alerts: content appropriateness and patient relevance in clinical practice
Source: Front Pharmacol. 2025 Mar 7;16:1510425. doi: 10.3389/fphar.2025.1510425 (PMC11925916; doi:10.3389/fphar.2025.1510425)
Supplement: Supplementary file 1 [file Supplementaryfile1.pdf]

Supplementary Material

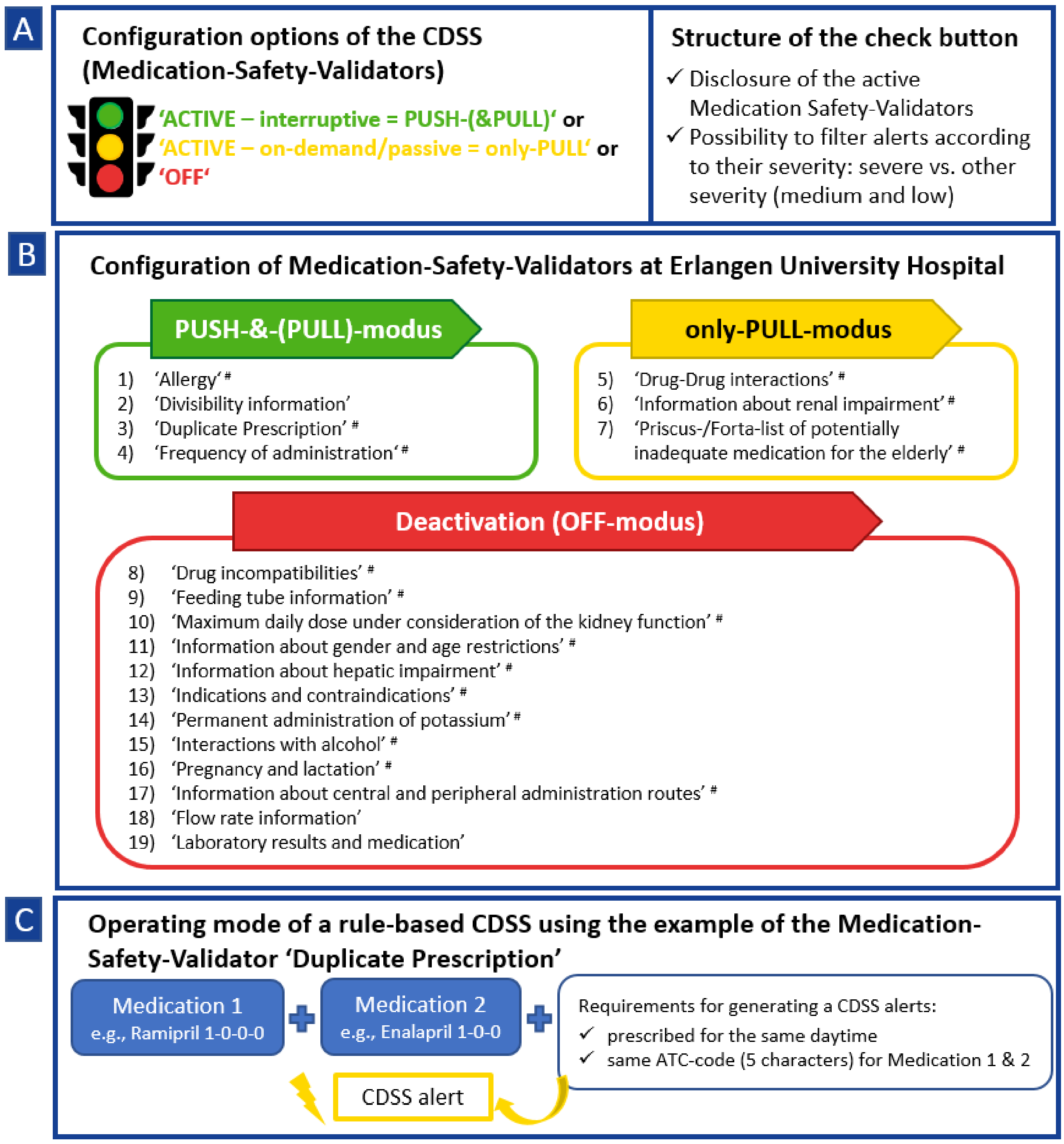

Supplementary Figure 1. Configuration options of (A) the Meona medication CDSS, (B) the selected configuration at Erlangen University Hospital, and (C) an example of the operating mode of the rule-based CDSS using the Medication-Safety-Validator 'duplicate prescription'. # Medication-Safety-Validators, for which CDSS-alerts are displayed by using the check button.*(Bauer et al., 2024)*

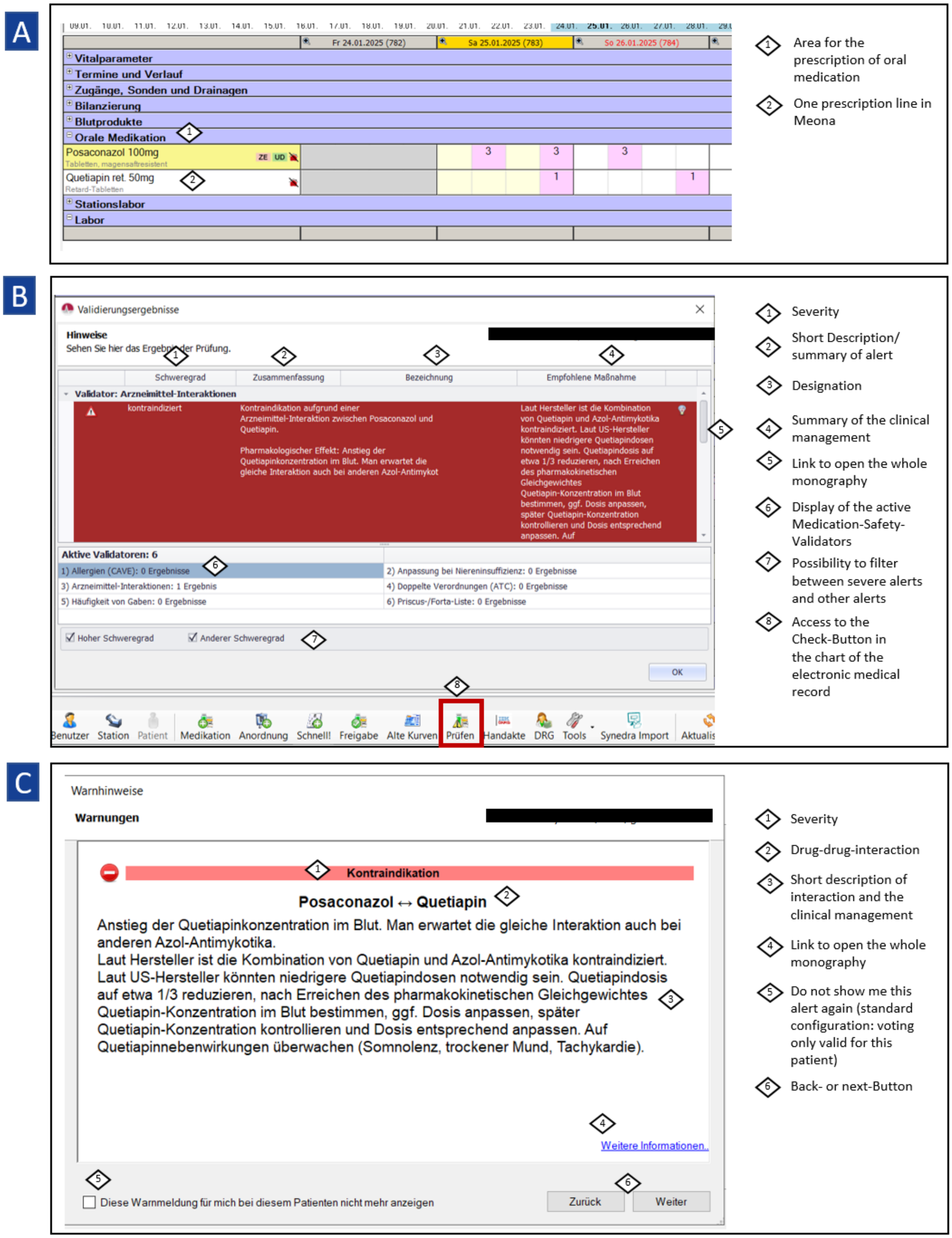

Supplementary Figure 2. (A) Example for the medication chart of Meona with two prescription lines, (B) the structure of the check button display, and (C) Example of one CDSS-PUSH-alert during medication prescription.

	Example content *in*appropriate	Example content appropriate	Example *not* patient relevant	Example patient relevant
**Allergy**	Documented: pollen allergy & Dekristol® (colecalciferol)	Documented: penicillin allergy & Piperacillin+Tazobactan	Patient relevance always depends on individual patient assessment, no general decision can be made	
**Duplicate prescription**	Ramipril+Hydrochlorothiazid 1-0-0-0 & Ramipril 0-0-0-1 (common combination in order to avoid nycturia)[#]	Ramipril&Enalapril<br><br>Clopidogrel&Aspirin	Clopidogrel&Aspirin (Common, concurrent use for a defined time)	Ramipril&Enalapril (Combination is not common and evidence-based)
**Information about renal impairment**	Aspirin 100mg and eGFR=45ml/min (low dose aspirin is safe in patients with renal impairment)	Sitagliptin + eGFR=35ml/min (dose adjustment might be necessary depending on the prescribed dose)<br><br>Apixaban + eGFR= 25ml/min (dose adjustment might be necessary depending on the prescribed dose)	Sitagliptin 50mg 1-0-0-0 & eGFR= 35ml/min (Already correct reduced dose)	Apixaban 5mg 1-0-1-0 & eGFR=25ml/min (Dose adjustment necessary)
**Drug-drug interaction**	Azithromycin&Nifedipin (no interaction in other databases such as UpToDate, Stockleys, and MediQ)	Ramipril&Spironolactone (known interaction: risk for hyperkalemia)<br><br>Ciprofloxacin&Ondansetron (ECG-monitoring required: QT-prolongation, both drugs = known risk according to credible meds)	Ramipril&Spironolactone & potassium level 3,9mmol/l (no presence of hyperkalemia)	Ciprofloxacin& Ondansetron (EKG-monitoring required: QT-prolongation, both drugs = known risk according to credible meds)
**PIM**	Zopiclone & Patient age = < 65 years (only inadequate medication for the elderly)[#]	Nifedipine as rescue medication for hypertension<br><br>Zopiclone & patient age = 85 years (potentially inadequate medication for the elderly)	Nifedipine on-demand as rescue medication (no better therapeutic alternatives available in Germany)	Zopiclone as sleeping pill (increased fall risk, better alternative e.g., melperone are available)

Supplementary Table 1. Examples of content inappropriate, content appropriate, not patient relevant, and patient relevant CDSS alert for every Medication-Safety-Validator, which is configured at Erlangen University. ‘Divisibility information’ and ‘Frequency of administration

were excluded, because no CDSS alert was displayed in our investigation. #Fictitious CDSS alert as an example, because none appeared, Abbreviations: ECG, electrocardiogram; eGFR, estimated glomerular filtration rate; PIM, potential inadequate medication in the elderly

Patient characteristics	**Number of patients TS (n=40)**	**Number of patients VS (n=40)**	**Number of patients M4 (n=40)**	**Number of patients M1 (n=40)**
Age in years median [range]	77.0 [11.0-94.0]	74.5 [60.0-88.0]	63.0 [21.0-91.0]	64.5 [29.0-105.0]
Age > 65 years number (%)	24 (60.0)	33 (82.5)	19 (47.5)	19 (47.5)
Prescription lines per patient median [range]	7.0 [2.0-26.0]	14.5 [4.0-27.0]	15.0 [4.0-32.0]	10.0 [2.0-20.0]
Use of long-term medication number (%)	32 (80.0)	40 (100.0)	39 (97.5%)	37 (92.5)
Polymedication * number (%)	19 (47.5)	33 (82.5)	32 (80.0)	20 (50.0)
Patients with renal impairment # number (%)				
eGFR ≥ 60ml/min/1.73 m²	31 (77.5)	23 (57.5)	2 (5.0)	29 (72.5)
eGFR 59-30ml/min/1.73 m²	7 (17.5)	9 (22.5)	6 (15.0)	9 (22.5)
eGFR < 30ml/min/1.73 m²	2 (5.0)	4 (10.0)	20 (50.0)	2 (5.0)
Dialysis	0 (0.0)	4 (10.0)	12 (30.0)	0 (0.0)

Supplementary Table 2. Patient characteristics for each clinical department. *Polymedication is defined as the use of at least five long-term medications. #Using CKD-EPI as an estimation for the GFR [glomerular filtration rate]. Abbreviations: M4, Medicine 4; M1, Medicine 1; TS, Trauma Surgery; VS, Vascular Surgery.

	M4 CDSS alerts (n=741)	M1 CDSS alerts (n=237)	TS CDSS alerts (n=268)	VS CDSS alerts (n=553)
**Number of CDSS alerts/patient median [range]**	16.0 [0.0-54.0]	4.0 [0.0-44.0]	3.0 [0.0-52.0]	14.0 [1.0-34.0]
**Number of CDSS alerts/prescription line**	1.3	0.58	0.74	0.99
**Number of CDSS alerts with high severity absolute**	220	87	91	211
**Number of CDSS alerts with high severity/patient median [range]**	4.5 [0.0-19.0]	1.5 [0.0-19.0]	1.0 [0-19]	5.0 [0-11]

Supplementary Table 3. Total number of CDSS alerts per patient and per prescription line for each clinical department. Abbreviations: M4, Medicine 4; M1, Medicine 1; TS, Trauma surgery; VS, Vascular Surgery; CDSS, clinical decision support system.

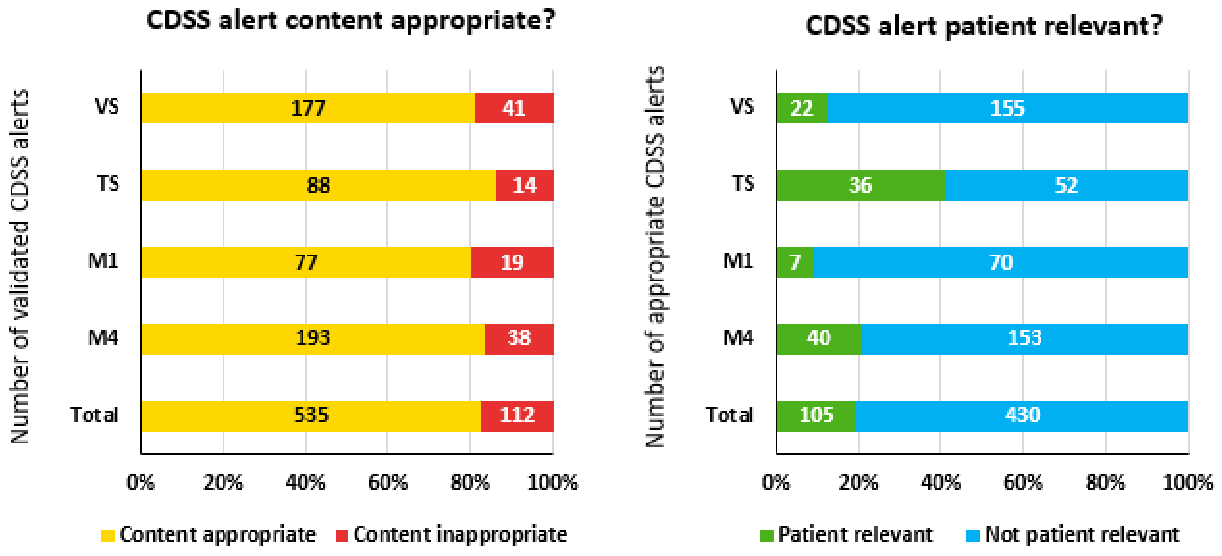

Supplementary Figure 3. Percentage of (A) content appropriate and (B) patient relevant CDSS alerts for the total validated CDSS alerts (N=647) and for each clinical department. Abbreviations: M4, Medicine 4; M1, Medicine 1; TS, Trauma surgery; VS, Vascular Surgery; CDSS, clinical decision support system.

**Alert types**	**Involved medication(-group)**	**Intervention**	***Example***
**Drug-drug interaction & drug-drug interaction**	Multiple antihypertensive drugs associated with hypotension	Pause antihypertensive drugs during hypotension	*Clonidin & Metoprolol and Metoprolol & Moxonidin*
	Multiple drugs associated with QT-prolongation	ECG control	*Amiodaron & Quetiapin and Amiodaron & Melperon*
	Multiple drugs associated with serotonin syndrome	Monitoring temperature, blood pressure, heart rate and for signs of seizures and tremor	*Escitalopram & Tramadol and Escitalopram & Fentanyl*
	Drugs that affect the absorption of other drugs	Delayed intake or complete discontinuation or replacement by more suitable alternatives	*Antacids (aluminium hydroxide) & mycophenolate and Antacids (magnesium hydroxide) & mycophenolate*
**Drug-drug interaction & information about renal impairment**	Drugs with additive risk for hyperkalemia and nephrotoxicity	Monitoring Potassium levels, pause/discontinue medication, dose adjustments	*Valsartan & Spironolacton and Valsartan (renal impairment) and Spironolacton (renal impairment)*
**Information about renal impairment & PIM**	Digoxin	TDM	*Digoxin (renal impairment) and Digoxin (PIM)*

Supplementary Table 4. Several patient-relevant CDSS alerts for one patient that concern the same drug-related problem and therefore only lead to one intervention. Abbreviations: PIM, potential inadequate medication in the elderly; TDM, therapeutic drug monitoring.

	M4 (n=32)	M1 (n=40)	TS (n=40)	VS (n=38)
**Number of total interventions**	77	30	63	74
**Number of total interventions/patient**	1.9	0.8	1.6	1.9
**CDSS-triggered interventions** number (%)	30 (39.0)	5 (16.7)	33 (52.4)	18 (24.3)
**Number of CDSS-triggered interventions/patient**	0.8	0.1	0.8	0.5
**CDSS-independent interventions** number (%)	47 (61.0)	25 (83.3)	30 (47.6)	56 (75.7)
**Number of CDSS-independent interventions/patient**	1.2	0.6	0.8	1.4

Supplementary Table 5. Number of pharmaceutical interventions (absolute and per patient) in total and divided into CDSS-triggered and CDSS-independent interventions (absolute, proportion, and per patient) for each clinical department. Abbreviations: M4, Medicine 4; M1, Medicine 1; TS, Trauma surgery; VS, Vascular Surgery; CDSS, clinical decision support system.

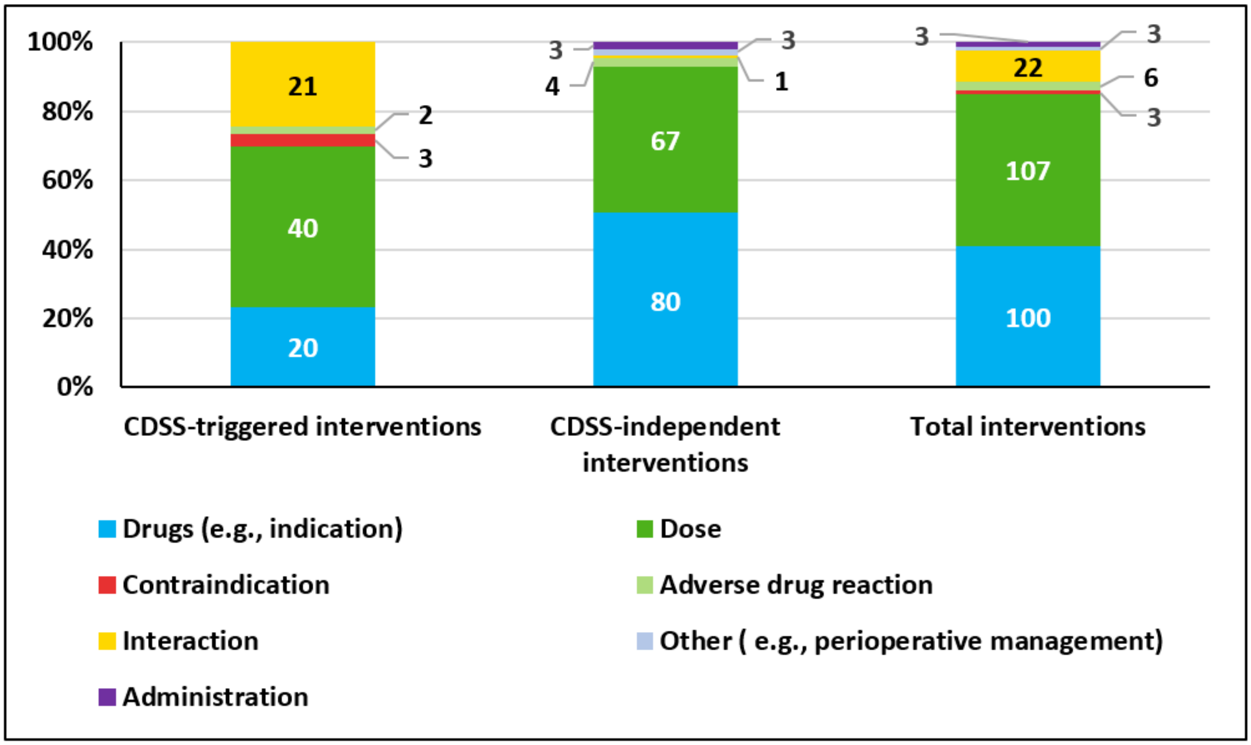

Supplementary Figure 4. Classification of reasons for pharmaceutical interventions divided into CDSS-triggered, CDSS-independent, and total interventions. Abbreviation: CDSS, clinical decision support system.

	Reasons and categories for interventions	CDSS-triggered interventions	CDSS-independent interventions
1	**Administration**	**0**	**3**
	Request/Query concerning administration/compatibility	0	0
	Administration (route)	0	3
	Administration (duration)	0	0
	Incompatibility or incorrect preparation/reconstitution	0	0
2	**Adverse drug reaction**	**2**	**4**
3	**Contraindication**	**3**	**0**
4	**Dose**	**40**	**67**
	Failure to adjust dose for organ dysfunction	34	11
	(Inappropriate) dose	0	20
	(Inappropriate) administration interval	0	14
	TDM not performed or neglected	6	22
5	**Drugs**	**20**	**80**
	(Clear) indication not (or no longer) given	0	12
	(Clear) indication, but no drug prescribed	0	36
	Drug allergy or medical history not considered	0	2
	Double prescription	14	11
	Wrong drug dispensed	0	0
	Generic/therapeutic substitution	0	0
	Transcription error	0	2
	Inappropriate (or not most suitable) drug formulation in terms of indication	0	3
	Inappropriate (or not most suitable) drug in terms of costs	0	0
	Inappropriate (or not most suitable) drug in terms of indication	2	9
	Prescription/documentation incomplete/incorrect	4	5
6	**Interaction**	**21**	**1**
7	**Other**	**0**	**3**
	Procurement/costs	0	0
	Advisory service/drug choice	0	1
	Advisory service/drug dose	0	1
	Advisory service/ Patients	0	0
	Failure to discontinue relevant drugs pre-/perioperatively	0	1

Supplementary Table 6. Absolute proportion of possible reasons (ordered in the ADKA-DokuPIK-categories) for the pharmaceutical interventions carried out. Abbreviation: TDM, therapeutic drug monitoring.

References

Bauer, J., Busse, M., Kopetzky, T., Seggewies, C., Fromm, M.F., and Dörje, F. (2024). Interprofessional Evaluation of a Medication Clinical Decision Support System Prior to Implementation. *Appl Clin Inform* 15(3)**,** 637-649. doi: 10.1055/s-0044-1787184.
